# Associations of symptoms of anxiety and depression with health-status, asthma control, dyspnoea, dysfunction breathing and obesity in people with severe asthma

**DOI:** 10.1186/s12931-022-02266-5

**Published:** 2022-12-12

**Authors:** Michelle A. Stubbs, Vanessa L. Clark, Peter G. Gibson, Janelle Yorke, Vanessa M. McDonald

**Affiliations:** 1 National Health and Medical Research Council Centre for Research Excellence in Severe Asthma, Level 2 West Wing, 1 Kookaburra Circuit, New Lambton Heights, NSW 2305 Australia; 2 Asthma and Breathing Research Centre, Hunter Medical Research Institute, Lot 1, Kookaburra Circuit, New Lambton Heights, NSW 2305 Australia; 3 School of Nursing and Midwifery, College of Health, Medicine and Wellbeing, The University of Newcastle, University Drive, Callaghan, NSW 2308 Australia; 4 Department of Respiratory and Sleep Medicine, John Hunter Hospital, New Lambton Heights, NSW 2305 Australia; 5 School of Health Sciences, University of Manchester, Oxford Road, Manchester, M13 9PL UK; 6 Christie Patient Centred Research, The Christie NHS Foundation Trust, Wilmslow Road, Manchester, M20 4BX UK

**Keywords:** Severe asthma, Anxiety, Depression, Quality of life, Asthma control, Dyspnoea, Dysfunctional breathing, Obesity

## Abstract

**Background:**

Anxiety and depression are comorbidities of severe asthma. However, clinical characteristics associated with coexisting severe asthma and anxiety/depression are poorly understood. The study objective is to determine clinical characteristics associated with anxiety and depressive symptoms in severe asthma.

**Methods:**

Severe asthma participants (N = 140) underwent a multidimensional assessment. Categorization of symptoms of anxiety and depression were based on HADS scale sub-scores and divided into four groups (< 8 on both subscales; ≥ 8 on one subscale; ≥ 8 on both subscales). Clinical characteristics were compared between subgroups. Multivariate logistic regression determined associations of clinical characteristics and anxiety and/or depressive symptoms in people with severe asthma.

**Results:**

Participants were (mean ± SD) 59.3 ± 14.7 years old, and 62% female. There were 74 (53%) severe asthma participants without symptoms of anxiety/depression, 11 (7%) with symptoms of anxiety, 37 (26%) with symptoms of depression and 18 (13%) with symptoms of anxiety and depression. Quality of life impairment was greater in participants with symptoms of depression (4.4 ± 1.2) and combined symptoms of anxiety and depression (4.4 ± 1.1). Asthma control was worse in those with symptoms of depression (2.9 ± 1.1) and combined anxiety and depression (2.6 ± 1.0). In multivariate models, dysfunctional breathing was associated with symptoms of anxiety (OR = 1.24 [1.01, 1.53]). Dyspnoea was associated with symptoms of depression (OR = 1.90 [1.10, 3.25]). Dysfunctional breathing (OR 1.16 [1.04, 1.23]) and obesity (OR 1.17 [1.00, 1.35]) were associated with combined symptoms of anxiety and depression.

**Conclusion:**

People with severe asthma and anxiety and/or depressive symptoms have poorer QoL and asthma control. Dyspnoea, dysfunctional breathing and obesity are associated with these symptoms. These key clinical characteristics should be targeted in severe asthma management.

## Background

Severe asthma occurs in approximately 3–8% of people with asthma [1, 2], yet the associated burden adversely impacts quality of life by a disproportionate amount [3]. Elevated levels of burden may be observed in people with severe asthma due to frequent acute attacks and multiple comorbidities [4]. In severe asthma, anxiety and depression are commonly associated comorbidities. A multicentre cross-sectional and two-year prospective cohort study reported 38% of participants with severe asthma express symptoms of anxiety and 25% express symptoms of depression, compared to 30% and 9%, respectively of a non-severe asthma population [5]. The presence of anxiety and depression also correlates with asthma control [6] and may increase the risk of acute asthma attacks ten Brinke et al. (2005), demonstrated an 11-fold increased risk for two or more acute attacks in people with severe asthma who experience comorbid anxiety and depression compared to those without [7].

Shared comorbidity in depressive and anxiety disorders is also common. This occurs due to the interplay of symptoms shared by both disorders, with overlapping symptoms acting as so-called bridges, funnelling symptom activation between symptom clusters of each disorder [8]. However, anxiety and depression can be reliably distinguished from each other [9], with diagnostic criteria designed to distinguish between disorders, and exclude clinical features that are common to more than one [10]. Thus, criteria for depression exclude common comorbid anxiety symptoms, and those for anxiety disorders exclude depressive symptoms. However, diagnostic criteria are not the same as clinical presentations [10]. Clinical presentations may be complicated by symptom covariation and lack of symptom specificity [11]. Complicating matters further is the fact that anxiety and depression are clearly not identical emotional states despite the set of common (non-specific) features noted in both disorders [12]. With this in mind, we are led to question whether there are differences in clinical characteristics observed in people with severe asthma experiencing either symptoms of anxiety, symptoms of depression, or combined symptoms of anxiety and depression. Answering this question will identify key characteristics that should be prioritised in management. Given the burden of psychological comorbidity in severe asthma and negative outcomes on asthma control, we aimed to determine the clinical characteristics associated with symptoms of anxiety or depression, compared to symptoms of both anxiety and depression so that psychological comorbidity can be easily identified.

In this study, we aimed to determine if clinical characteristics associated with symptoms of anxiety and/or depression in a cohort of participants with severe asthma differed between groups, defined using a validated screening tool, the Hospital Anxiety and Depression Scale (HADS). Second, we aimed to determine associations between clinical characteristics, Health-Related Quality of Life (HRQoL) and asthma control with symptoms of anxiety or depression, as well as combined symptoms of anxiety and depression. First, we hypothesized that clinical characteristics would be different between participants with severe asthma and coexisting symptoms of anxiety and/or depression compared to those without. Secondly, we hypothesized that severe asthma and co-existing symptoms of anxiety and/or depression would be associated with increased levels of HRQoL impairment and poor asthma control.

## Methods

### Study design

Between July 2012 and October 2016, participants with severe asthma (N = 140) were recruited to a cross-sectional study to undertake a multidimensional assessment as part of the screening process prior to entering a randomised control trial which aimed to apply multidimensional assessment to define the number and type of traits present in a severe asthma population [13]. The assessment was conducted over two visits; characteristics/traits were characterised in the pulmonary, extrapulmonary and risk factor/ behavioural domains. The multidimensional assessment has been described elsewhere [14].

Adults with severe asthma were recruited concurrently from respiratory ambulatory care clinics at John Hunter Hospital (Newcastle, New South Wales, Australia), and clinical research databases of the Department of Respiratory and Sleep Medicine at the John Hunter Hospital (Newcastle, New South Wales, Australia). John Hunter Hospital is a tertiary public hospital, providing a range of medical and surgical services to a population of more than 800,000 people [15]. John Hunter Hospital is positioned within the Hunter New England Local Health District and covers a region of 131,785 square kilometres. It encompasses a major metropolitan centre and regional communities. All study visits were completed at the Hunter Medical Research Institute, Newcastle, New South Wales, Australia.

Prior to the commencement of the study, ethics approval was granted from Hunter New England Local Health District Human Research Ethics Committee (08/08/20/3.10). The study was conducted in accordance with Good Clinical Practice Guidelines and ethical principles consistent with the Declaration of Helsinki. All participants provided written informed consent, with investigators obtaining signed and dated consent forms.

### Participants

Adults with confirmed doctor diagnosed asthma and previous evidence of variable airflow limitation (previous evidence of bronchodilator response ≥ 200 mL or 12% post-bronchodilator FEV_1_ following administration of 400 µg salbutamol); or airway hyper-responsiveness in response to any standard challenge agent; or peak flow variability (diurnal; variation ≥ 15% or ≥ 50 mL) were enrolled [1]. Additional inclusion criteria to establish a severe asthma diagnosis included: *(1)* administration of high dose inhaled corticosteroids (ICS) ≥ 1000mcg with long-acting beta-agonists or *(2)* maintenance prednisone and *(3)* Forced Expiratory Volume in one second (FEV_1_) post bronchodilator: < 80% Predicted or FEV_1_/Forced Volume Capacity (FVC) < 70%; or *(4)* Asthma Control Questionnaire (ACQ7) score ≥ 1.5 [15]; or *(5)* severe exacerbation within the previous 12 months requiring oral corticosteriods (OCS) [1]. Exclusion criteria included non-English speaking, being aged < 18 years and inabliity to attend study visits, diagnosed with current lung cancer, lymphatic or solid organ malignancy or had a poor prognosis (< 3 months life expectancy).

### Clinical measures

Participants underwent a multidimensional assessment to identify clinical characteristics, previously described [14]. Multidimensional assessment of pulmonary characteristics involved spirometry (forced expiratory volume in one second (FEV_1_%) predicted, forced vital capacity (FVC%) predicted and FEV_1_/FVC%) post bronchodilator therapy [16]; airway T2 high inflammation [fractional exhaled nitric oxide (FeNO)] [17]; induced sputum to assess airway inflammation (sputum eosinophil and neutrophil proportions); dyspnoea (modified Medical Research Council (mMRC) dyspnoea score). Multidimensional assessment of extra-pulmonary characteristics involved blood collection to assess systemic inflammatory markers (high sensitivity C reactive protein mg/L (hs-CRP), interleukin (IL)-6 pg/mL, full blood count for blood eosinophil and neutrophil counts × 10^9^/L); atopic status (serum IgE), dysfunctional breathing (Nijmegen score) [18] and obesity (BMI). Multidimensional assessment of risk factors and behavioural characteristics involved the absence of a written asthma action; exercise capacity (six-minute walk distance [6MWD]) [19].

Additional assessment measures included anxiety and depressive symptoms [Hospital Anxiety and Depression Scale (HADS)] [20], Health-Related Quality of Life (Asthma Quality of Life Questionnaire [AQLQ]) [21]; and asthma control (Asthma Control Questionnaire [ACQ7]) [15].

### Statistical analysis

Statistical analysis was performed using STATA v.15 software. Data are reported as means (SD) and medians (Q_1_, Q_3_). Student’s t-test or the two-sample Wilcoxon rank sum test was used as appropriate for group comparisons. ANOVA or the Kruskal–Wallis test was used for more than two groups with Bonferroni correction. Categorical data were analyzed using the Fisher’s exact test, with the Fisher’s p-value reported when expected counts are < 5. Associations were determined using simple and multivariate logistic regression in addition to the Spearman’s correlation to determine strength and direction. Simple logistic regression (adjusted for age and sex) was performed to identify characteristics to include in a multivariate model, characteristics were included when p < 0.2, additional variables such as FEV_1_% predicted, asthma control, quality of life, age and sex were additionally added to the model as they were considered clinically relevant to the outcome. Significance was accepted when p < 0.05.

Groups were determined using the HADS, symptoms of anxiety and depression were classified using a score of ≥ 8 on the respective anxiety or depression subscales. Groups are classified as severe asthma without either anxiety or depression (severe asthma no A and/or D), with symptoms of anxiety (severe asthma + A), severe asthma with symptoms of depression (severe asthma + D) and severe asthma with combined symptoms of anxiety and depression (severe asthma + A&D).

Based on our sample size of N = 140 we had 90% power to detect population associations of approximately f^2^ = 0.15 (medium effect size) for 9 predictor variables, using 0.05 level tests.

## Results

### Clinical characteristics of anxiety and/or depression

Participants’ (N = 140) demographics, pulmonary, extra-pulmonary, risk-factors and behavioural characteristics are presented in Table 1 according to groupings.

**Table 1:** Baseline participant characteristics

Characteristic	Measure	Sample population	Severe asthma with No Anxiety and/or Depression (severe asthma noAand/orD)	Severe asthma with Anxiety (severe asthma + A)	Severe asthma with Depression (severe asthma + D)	Severe asthma with both Anxiety and Depression (severe asthma + A&D)
Demographics						
Sample number	n	140	74	11	37	18
Female	n (%)	87 (62)	39 (53)	6 (55)	27 (73)	15 (83)^✦^*
Age	Years, median [Q1,Q3]	59.3 [18.6, 82.3]	60.5 [23.9, 78.8]	68.5 [18.9, 76.2]	52.8 ([8.6, 73.5]	54.9 ([3.3, 82.3]
OCS daily use						
Yes	n (%)	40 (29)	20 (27)	5 (45)	10 (27)	5 (28)
No		100 (71)	54 (73)	6 (54)	27 (72)	13 (72)
OCS dose	mg/day	14 (1, 50)	11 (1, 50)	19 (1, 50)	15 (5, 25)	21 (2, 30)
ICS dose	Beclomethasone equv. µg/day	2000 (2000, 2000)	2000 (2000, 2000)	2000 (2000, 2000)	2000 (2000, 2000)	2000 (2000, 2000)
Total exacerbation past year	median, range	3.1 (0, 15)	2.5 (0, 12)	3.5 (0, 15)	4.4 (0, 14)^✦^*	2.9 (0, 10)
Current antidepressant/anxiolytic use	n (%)	33 (24)	20 (27)	4 (36)	18 (49)	3 (17)
Pulmonary						
Airflow limitation	FEV_1_% predicted, mean [95% CI]	74.7 [71.2, 78.3]	75.1 [70.3, 79.7]	66.3 [48.8, 83.8]	74.7 [67.5, 82.0]	79.0 [68.7, 89.3]
	Post β_2_ FEV_1_/FVC %, mean [95% CI]	85.0 [82.2, 87.7]	86.8 [83.3, 90.3]	75.9 [60.9, 90.9]	83.4 [77.8, 89.0]	86.1 [78.2, 94.1]
	Post β_2_ FVC % predicted, mean [95% CI]	68.0 [65.7, 70.2]	66.5 [63.4, 69.6]	66.9 [55.8, 78.1]	69.4 [65.0, 73.7]	71.8 [66.3, 77.4]
Airway T2 high inflammation	FeNO (ppb), mean [95% CI]	28.0 [22.2, 33.8]	28.2 [20.2, 36.2]	15.3 [7.3, 23.3]	32.8 [20.0, 45.6]	23.9 [3.9, 44.0]
Airway inflammation—eosinophilic	Sputum Eosinophils (%), mean [95% CI]	8.6 [6.4, 10.8]	9.5 [6.0, 13.0]	4.6 [− 0.4, 9.6]	8.4 [4.8, 12.0]	7.4 [1.4, 13.3]
Airway inflammation—neutrophilic	Sputum Neutrophils (%), mean [95% CI]	40.0 [35.5, 44.5]	40.1 [34.2, 46.0]	47.8 [30.3, 65.2]	40.5 [30.3, 50.7]	34.2 [20.0, 48.3]
Dyspnoea	MMRC Score, mean [95% CI]	2.0 [1.8, 2.3]	1.6 [1.3, 1.8]	2.2 [1.2, 3.1]	2.6 [2.2, 3.0]^✦^***	2.8 [2.2, 3.4]^✦^***
Extra pulmonary						
Dysfunctional breathing	Nijmegen Score, mean [95% CI]	22.6 [20.7, 24.6]	18.6 [16.1, 21.0]	25.1 [15.7, 34.5]	25.9 [22.4, 29.4]^✦^*	31.1 [26.6, 35.6]^✦^***
Systemic inflammation	Hs-CRP (mg/L), mean [95% CI]	6.9 [4.6, 9.3]	3.5 [2.4, 4.6]	17.8 [-9.7, 45.2]	10.8 [6.0, 15.5]^✦^***	6.4 [2.7, 10.1]
	IL-6 (ng/mL), mean [95% CI]	3.5 [2.7, 4.3]	3.1 [2.3, 3.8]	3.2 [0.9, 5.5]	4.4 [2.5, 6.4]	3.3 [0.5, 6.1]
	Blood Eosinophils (× 10^9^/L), mean [95% CI]	0.3 [0.2, 0.3]	0.3 [0.2, 0.3]	0.3 [0.1, 0.4]	0.3 [0.2, 0.4]	0.4 [0.2, 0.6]
	Blood Neutrophils (× 10^9^/L), mean [95% CI]	5.3 [4.9, 5.8]	5.0 [4.5, 5.5]	6.4 [3.8, 9.0]	6.1 [5.2, 6.9]	4.5 [3.7, 5.3]^*
Atopic status	IgE (IU/mL), mean [95% CI]	415.5 [280.4, 550.5]	432.7 [252.3, 613.1]	216.3 [47.4, 385.2]	349.2 [103.9, 594.6]	587.1 [− 1.9, 1176.1]
Obesity	BMI (kg/m^2^), mean [95% CI]	31.6 [30.2, 32.9]	30.4 [28.9, 31.9]	28.7 [22.9, 34.4]	32.3 [29.6, 35.1]	36.8 [30.8, 42.8]^✦^*
Risk factors and behavioural						
Absence of written action plan	Absence of WAP, n (%)	44 (31)	21 (28)	2 (18)	13 (35)	8 [44]
Exercise intolerance	6-min walk test (metres), mean [95% CI]	452.4 [434.4, 470.4]	469.4 [445.0, 493.8]	454.8 [374.4, 535.3]	439.1 [403.9, 474.2]	404.3 [352.2, 456.5]

Descriptive data presented as n (%), mean ± SD or median [Q1, Q3]. Bonferroni post hoc test and Kruskal Wallis post hoc Test: ^✦^p < 0.02 versus severe asthma only participants; ^ = versus Severe asthma + D. Levels of significance: * < 0.05; ** < 0.01; *** < 0.001; **** < 0.0001. OCS (Oral Corticosteroids), ICS (Inhaled Corticosteroids), FEV1 (Forced Expiratory Volume in one second), FVC (Forced Volume Capacity), FeNO (Fractional exhaled Nitric Oxide), Ppb (Parts per billion), mMRC (Modified Medical Research Council), BMI (Body Mass Index), hs-CRP (High sensitivity-C-Reactive Protein), IL-6 (Interleukin), IgE (Immunoglobulin), 6MWD (6-Minute Walk Distance), WAP (Written Action Plan)

### Demographic characteristics

Participants were mostly female, overweight, middle aged and prescribed high dose inhaled corticosteroids (Table 1). Overall participants experienced at least 2 exacerbations in the past year, with approximately one quarter of the overall study population currently using antidepressant/anxiolytic medications (Table 1). Sex was significantly different between subgroups with a higher proportion of females in the severe asthma + A & D (15 [83.3%]) compared to severe asthma noAand/orD (39 [52.7%], *p* = 0.03) (Table 1). Total exacerbation frequency over the past 12 months was also significantly different between severe asthma + D (4.4 [0, 14]) compared to severe asthma noAand/orD (2.5 [0, 12], *p* = 0.04) (Table 1). Whilst a proportion of participants were prescribed maintenance OCS (29%, N = 40) and anxiolytic/antidepressant medications (24%, N = 33) (Table 1), there were no significant differences between groups.

### Pulmonary characteristics

Across all groups, there were no significant differences in measures of airflow limitation or FeNO (Table 1). FEV1% predicted was lower in the Severe asthma + A group than all other groups, however this difference is not significant. Sputum neutrophil and eosinophil proportions were not statistically different across groups (Table 1). This finding was further explored (Table 2) to determine if anxiety or depression or a combination of both were related to specific airway inflammatory phenotypes. Our results demonstrated that neither symptom of anxiety and depression alone or combined are associated with any asthma airway inflammatory phenotype (Table 2). Dyspnoea indicated by mMRC scores were significantly higher in severe asthma + A & D (2.8 [2.2, 3.4], *p* = 0.0007) and severe asthma + D (2.6 [2.2, 3.0] *p* = 0.0002) compared to severe asthma no A and/or D (1.6 [1.3, 1.8]) (Table 1). Dysfunctional breathing indicated by Nijmegen scores were significantly higher in severe asthma + A & D (31.1 [26.6, 35.6], *p* = 0.0001) and severe asthma + D (25.9 [22.4, 29.4], *p* = 0.004) compared to severe asthma noAand/orD (18.6 [16.1, 21.0) (Table 1).

**Table 2:** Associations of airway and systemic inflammation by symptoms of anxiety, depression and combined anxiety and depression compared to severe asthma with no anxiety and/or depression

	Severe asthma with Anxiety (Severe asthma + A) compared to Severe asthma with No Anxiety and/or Depression (severe asthma no A and/or D)			Severe asthma with Depression (Severe asthma + D) compared to Severe asthma with No Anxiety and/or Depression (severe asthma no A and/or D)			Severe asthma with both Anxiety and Depression (Severe asthma + A & D) compared to Severe asthma with No Anxiety and/or Depression (severe asthma no A and/or D)		
	Yes	No	p-value	Yes	No	p-value	Yes	No	p-value
Airway									
Sputum Eosinophil									
Low < 3%	5 (55.6)	29 (46.8)	0.62	14 (46.7)	29 (46.8)	0.99	6 (40.0)	29 (46.8)	0.64
High > 3%	4 (44.4)	33 (53.3)		16 (53.3)	33 (53.2)		9 (60.0)	33 (52.2)	
Sputum Neutrophil									
Low < 60%	6 (66.7)	45 (72.6)	0.71	22 (73.3)	45 (72.6)	0.94	13 (86.7)	45 (72.6)	0.26
High > 60%	3 (33.3)	17 (27.4)		8 (26.7)	17 (27.4)		2 (13.3)	17 (27.4)	
Systemic									
hs-CRP									
Low < 3 mg/L	3 (30.0)	48 (69.6)	**0.02**	12 (32.4)	48 (69.6)	**< 0.001**	7 (41.2)	48 (69.6)	**0.03**
High > 3 mg/L	7 (70.0)	21 (30.4)		25 (67.6)	21 (30.4)		10 (58.8)	21(30.43)	

Categorical data presented as n (%) of proportions. Bolding indicates significant p-value < 0.05 (Chi-Square test). hs-CRP (High sensitivity-C-Reactive Protein)

### Extra-pulmonary characteristics

Significant differences between severe asthma no A and/or D (30.4 [28.9, 31.9], p = 0.04) and severe asthma + A & D (30.6 [30.8, 42.8],) in terms of obesity were found (Table 1). Compared to severe asthma noAand/orD, hs-CRP levels were significantly higher in severe asthma + D (10.8. [6.0, 15.5]), but not significantly different in severe asthma + A&D (6.4 [2.7, 10.1]) and severe asthma + A (17.8 [− 9.7, 45.2], p = 0.0002) (Table 1).

Systemic inflammatory biomarkers including IL-6, blood eosinophils and IgE showed no significant differences across groups. Blood neutrophil cell counts were significantly lower in severe asthma + A & D (4.5 [3.7, 5.3) compared to severe asthma + D (6.1 [5.2, 6.9], p = 0.04) (Table 1). This finding was further explored (Table 2) to determine if anxiety or depression or a combination of both were related to systemic inflammation. We found a greater proportion of participants with either symptoms of anxiety, symptoms of depression or combined symptoms of anxiety and depression had increased levels of hs-CRP (Table 2).

### Risk factors and behaviours

Across all groups, there were no significant differences in the presence of written action plans (WAPs), however the proportion of people with a WAP was highest in severe asthma + A & D (8 [44]) and severe asthma + D (13 [35]) compared to severe asthma noAand/orD (21 [28]) and severe asthma + A (2 [18]). Participants with severe asthma + A & D walked fewer metres on the 6MWD compared to severe asthma + D, severe asthma + A and severe asthma no A and/or D, however this difference was not significant (Table 1).

### Health-related quality of life and asthma control

**Fig. 1:**
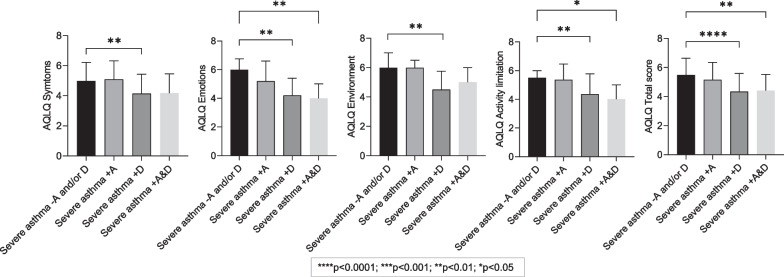
Baseline participant characteristics of AQLQ domains and total score. Descriptive data presented as n (%), mean ± SD. Bonferroni post hoc test and Kruskal Wallis post hoc Test. Groups are classified as severe asthma without either anxiety or depression (severe asthma-A and/or D), with symptoms of anxiety (severe asthma + A), severe asthma with symptoms of depression (severe asthma + D) and severe asthma with combined symptoms of anxiety and depression (severe asthma + A & D)

Overall HRQoL and asthma control was poor. HRQoL impairment was higher in severe asthma + A & D and severe asthma + D compared to severe asthma + A and severe asthma no A and/or D as indicated by AQLQ total questionnaire scores, with a significance difference between severe asthma + A & D (4.4 ± 1.1), severe asthma + D (4.4 ± 1.2), versus severe asthma noAand/orD (5.5 ± 1.2, *p* < 0.0001) (Fig. 1). In terms of AQLQ domains, impairment was highest in severe asthma + D and severe asthma + A&D compared to severe asthma + A and severe asthma no A and/or D (Fig. 1). AQLQ symptom and environmental domain scores were statistically significant in severe asthma + D compared to severe asthma no A and/or D (Fig. 1). AQLQ activity limitation and emotional function domains were statistically significant between severe asthma + D, and severe asthma + A & D compared to severe asthma no A and/or D (Fig. 1).

**Fig. 2:**
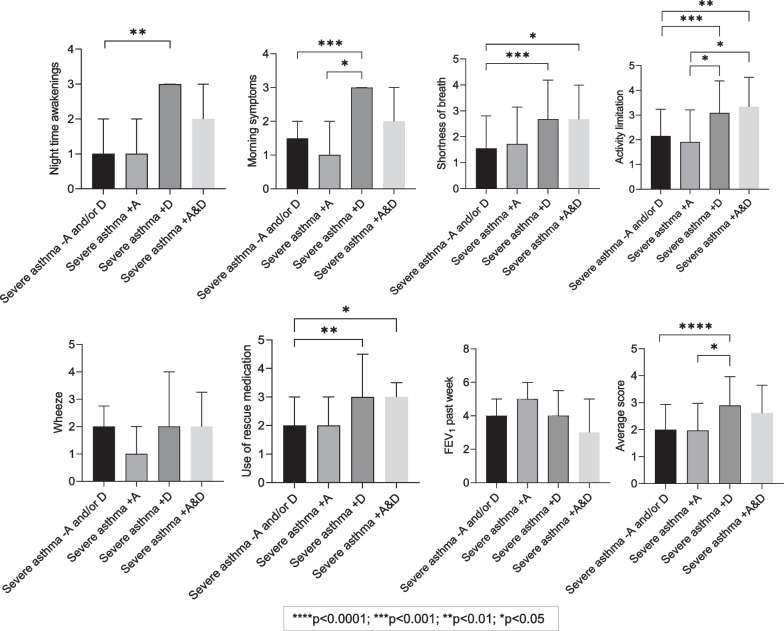
Baseline participant characteristics of asthma control and average score. Descriptive data presented as n (%), mean ± SD. Bonferroni post hoc test and Kruskal Wallis post hoc Test. Groups are classified as severe asthma without either anxiety or depression (severe asthma-A and/or D), with symptoms of anxiety (severe asthma + A), severe asthma with symptoms of depression (severe asthma + D) and severe asthma with combined symptoms of anxiety and depression (severe asthma + A & D)

Asthma control, defined by average ACQ7 scores, was worse in severe asthma + D and severe asthma + A & D compared to severe asthma + A and severe asthma no A and/or D, with a significance difference between severe asthma + D versus severe asthma no A and/or D (2.9 ± 1.1 and 2.0 ± 0.9, *p* < 0.0001) and between severe asthma + D versus severe asthma + A (2.9 ± 1.1 and 2.0 ± 1.0, *p* = 0.04) (Fig. 2). An analysis of AQLQ and ACQ by items/domains is presented in Figs. 1 and 2 respectively.

### Anxiety and/or depression in severe asthma are associated with few clinical characteristics

The regression models examining the associations of symptoms of anxiety or depression alone and combined symptoms of anxiety and depression are shown in Tables 3, 4 and 5. Multivariate logistic regression analysis of symptoms of anxiety (Table 3), showed that for every unit increase in Nijmegen score, there was a 1.24-fold ([95%CI 1.01, 1.53], *p* = 0.04) increase in the odds of experiencing symptoms of anxiety.

**Table 3:** Multiple logistic regression model for symptoms of anxiety (Model A)

Clinical characteristic determinants of psychological distress		
	Simple logistic regression	Multivariate logistic regression (Adjusted) r^2^ = 0.22

Clinical characteristic determinants of Anxiety model (A)				
Clinical characteristics	OR (95% CI)	p-value	Adjusted OR (95% CI)	p-value
Sex (female)	1.08 (0.30, 3.84)	0.91	0.12 (0.01, 1.95)	0.14
Age (years)	1.01 (0.97, 1.06)	0.66	0.97 (0.88, 1.06)	0.48
Airflow limitation (FEV_1_% predicted)	0.98 (0.95, 1.01)	0.17	0.91 (0.85, 0.99)	**0.03**
Dyspnoea (mMRC, score)	1.51 (0.91, 2.51)	0.11	1.95 (0.54, 7.10)	0.31
Dysfunctional breathing (Nijmegen, score)	1.05 (0.99, 1.11)	0.08	1.24 (1.01, 1.53)	**0.04**
Systemic inflammation (hs-CRP, mg/L)	1.07 (0.97, 1.19)	0.19	1.04 (0.91, 1.18)	0.58
Exercise tolerance (6MWD, metres)	0.99 (0.99, 1.00)	0.12	0.99 (0.99, 1.00)	0.40
Obesity (BMI, kg/m^2^)	1.04 (0.99, 1.09)	0.14	1.04 (0.98, 1.10)	0.20
Asthma control (ACQ, score)	0.97 (0.90, 1.10)	0.94	0.65 (0.42, 1.02)	0.06
Asthma Quality of Life (AQLQ, score)	0.93 (0.52, 1.66)	0.81	0.42 (0.04, 4.09)	0.45

Simple logistic regression was performed to identify multivariate characteristics (p < 0.2). Additional variables such as asthma control, quality of life, age and sex were additionally added to the models as they were considered clinically relevant to the outcome. mMRC (Modified Medical Research Council), hs-CRP (High sensitivity-C-Reactive Protein), 6MWD (6-Minute Walk Distance), BMI (Body Mass Index), ACQ (Asthma Control Questionnaire), AQLQ (Juniper Asthma Quality of Life Questionnaire). Bold font indicates significant p-value < 0.05

**Table 4:** Multiple logistic regression model for symptoms of depression (Model B)

Clinical characteristic determinants of psychological distress		
	Simple Logistic regression	Multivariate logistic regression (Adjusted) r^2=^0.26

Clinical characteristic determinants of Depression model (B)				
Clinical characteristic	OR (95% CI)	p-value	Adjusted OR (95% CI)	p-value
Sex (female)	2.42 (1.03, 5.71)	**0.04**	1.19 (0.37, 3.81)	0.77
Age (year)	0.98 (0.96, 1.01)	0.21	1.01 (0.96, 1.06)	0.76
Dyspnoea (mMRC, score)	2.00 (1.41, 2.87)	**< 0.01**	1.90 (1.10, 3.25)	**0.02**
Dysfunctional breathing (Nijmegen, score)	1.07 (1.03, 1.11)	**< 0.01**	1.02 (0.95, 1.08)	0.60
Systemic inflammation (hs-CRP, mg/L)	1.13 (1.04, 1.23)	**< 0.01**	1.09 (0.99, 1.20)	0.08
Exercise tolerance (6MWD, metres)	1.00 (0.99, 1.00)	0.16	1.00 (0.99, 1.01)	0.68
Obesity (BMI, kg/m^2^)	1.04 (0.98, 1.10)	0.19	1.00 (0.92, 1.10)	0.94
Asthma control (ACQ, score)	1.45 (1.07, 1.22)	**< 0.01**	1.11 (0.98, 1.25)	0.11
Asthma Quality of Life (AQLQ, score)	0.51 (0.35, 0.75)	**< 0.01**	1.05 (0.44, 2.51)	0.91

Simple logistic regression was performed to identify multivariate characteristics (p < 0.2). Additional variables such as asthma control, quality of life, age and sex were additionally added to the models as they were considered clinically relevant to the outcome. mMRC (Modified Medical Research Council), hs-CRP (High sensitivity-C-Reactive Protein), 6MWD (6- Minute Walk Distance), BMI (Body Mass Index), ACQ (Asthma Control Questionnaire), AQLQ (Juniper Asthma Quality of Life Questionnaire). Bold font indicates significant p-value < 0.05

**Table 5:** Multiple logistic regression model for symptoms of combined anxiety and depression (Model C)

Clinical characteristic determinants of psychological distress		
	Simple logistic regression	Multivariate logistic regression (Adjusted) r^2^ = 0.37

Clinical characteristic determinants of Combined Anxiety and Depression model (C)				
Clinical characteristic	OR (95% CI)	p-value	Adjusted OR (95% CI)	p-value
Sex (female)	4.49 (1.20, 16.81)	**0.03**	2.84 (0.44, 18.14)	0.27
Age (years)	0.98 (0.95, 1.01)	0.25	0.94 (0.88, 1.01)	0.08
Dyspnoea (mMRC, score)	2.37 (1.45, 3.88)	**< 0.01**	1.51 (0.63, 3.76)	0.35
Dysfunctional breathing (Nijmegen, score)	1.12 (1.05, 1.20)	**< 0.01**	1.16 (1.04, 1.23)	**0.01**
Systemic inflammation (hs-CRP, mg/L)	1.09 (0.99, 1.19)	0.07	0.87 (0.72, 1.04)	0.12
Exercise tolerance (6MWD, metres)	0.99 (0.99, 1.00)	**0.03**	1.00 (0.99, 1.01)	0.57
Obesity (BMI, kg/m^2^)	1.10 (1.02, 1.19)	**0.01**	1.17 (1.00, 1.35)	**0.04**
Asthma control (ACQ, score)	1.10 (1.02, 1.20)	**0.02**	0.98 (0.82, 1.18)	0.85
Asthma Quality of Life (AQLQ, score)	0.47 (0.28, 0.79)	**< 0.01**	1.53 (0.51, 4.64)	0.45

Simple logistic regression was performed to identify multivariate characteristics (p < 0.2). Additional variables such as asthma control, quality of life, age and sex were additionally added to the models as they were considered clinically relevant to the outcome. mMRC (Modified Medical Research Council), hs-CRP (High sensitivity-C-Reactive Protein), 6MWD (6- Minute Walk Distance), BMI (Body Mass Index), ACQ (Asthma Control Questionnaire), AQLQ (Juniper Asthma Quality of Life Questionnaire). Bold font indicates significant p-value < 0.05

For symptoms of depression (Table 4), the model showed that for every unit increase in mMRC score, there was a 1.90-fold ([95%CI 1.10, 3.25], *p* = 0.02) increase in the odds of experiencing symptoms of depression.

Dysfunctional breathing and obesity were associated with combined symptoms of anxiety and depression (Table 5). For every unit increase in Nijmegen score, there was a 1.15-fold ([95%CI 1.04, 1.23], *p* = 0.01) increase in odds of experiencing symptoms of combined anxiety and depression. Furthermore, for every 1 kg/m^2^ increase in BMI, there was a 1.17-fold ([95%CI 1.00, 1.35], *p* = 0.04) increase in the odds of experiencing symptoms of combined anxiety and depression (Table 5).

## Discussion

In this study we report that symptoms of anxiety and/or depression are present in almost 50% of people with severe asthma. We also report clinical characteristics that are associated with symptoms of either anxiety or depression, as well as symptoms of both anxiety and depression in a severe asthma population. These characteristics include dyspnoea, which is associated with symptoms of depression, and dysfunctional breathing which is associated with symptoms of anxiety. In the presence of symptoms of both anxiety and depression, we found that dysfunctional breathing and obesity were related. We also confirm the burden of anxiety and/or depressive symptoms, reporting their significant associations with impaired HRQoL and poor asthma control. These important data highlight the need to improve assessment and treatment of anxiety and/or depressive symptoms in the management of people with severe asthma to improve health-status. They also highlight the complexity of managing extra-pulmonary characteristics related to severe asthma through a significant inter-relationship between symptoms of anxiety, depression, dyspnoea and dysfunctional breathing.

Previous studies report underdiagnosis and undertreatment of anxiety and depression in chronic obstructive pulmonary disease (COPD) [22, 23]. Our data suggests this may also be the case in severe asthma, as 47% of our population had symptoms of anxiety or depression or both, but only 9% were currently treated with anxiolytic or antidepressant medication. Underdiagnosis is also identified by Valença and colleagues who report only 6.5% of outpatients with mild/moderate or severe asthma were undergoing treatment for anxiety and depression [24]. Underdiagnosis may be related to symptom misattribution and the co-existence of numerous confounding comorbidities in severe asthma.

We found that dyspnoea, obesity and dysfunctional breathing to be associated with symptoms of anxiety and/or depression, but the causal path of these symptoms is unknown and disentangling these symptoms is difficult for both patients and clinicians. For example, chest tightness is a key symptom in anxiety, severe asthma and is observed during acute attacks [25]. Adding to challenges surrounding symptom misattribution are asthma medications such as β-agonists that are known to be a primary cause of tachycardia. An increase in heart rate stemming from β-agonist use results from dilation of peripheral vasculature that reduces venous return, resulting in sympathetic nervous system reflexes and increased inotropic and chronotropic effects [26]. However, the same described symptom of tachycardia is characterized as a symptom of anxiety [27] that potentially results in elevated psychological testing scores. To avoid underdiagnosis and symptom misattribution, we suggest routine screening of psychological symptoms in severe asthma.

We found measures of dyspnoea and dysfunctional breathing to be significantly increased in people who experienced either anxiety or depression alone, and in those with both anxiety and depression compared to severe asthma only. Whilst minimal data are reported in severe disease, associations between anxiety, depression and increased dyspnoea has been reported in asthma studies of mild to moderate disease [28, 29]. Symptoms related to anxiety and depression have also been reported to be important determinants for the development of dyspnoea [30]. Anxiety and depression could be more easily understood as secondary to dyspnoea because of the burden dyspnoea brings to maintaining quality of life (QoL), however it is unknown if this is the case [31]. Our results may be explained by understanding dyspnoea as a two-dimension phenomenon—sensory and affective [32]. The subjective and multifactorial mechanisms of dyspnoea that include derangements of mechanical loads caused by increased airway resistance and hyperinflation, gas exchange abnormalities, and afferent mismatch [33] as well as, emotional and cognitive factors [31] may play part in anxiety and depression. Research in general populations suggests a ‘vicious circle’ in which dyspnoea causes anxiety and vice versa due to hyperventilation [31]. In terms of depression, emotional states are powerful modulators of the perception of dyspnoea [34]. This has been demonstrated in people with asthma, where strong emotions (both positive and negative) decrease pulmonary function [34]. Furthermore, depression could be part of pathophysiological respiratory sensations via the link between depression and inflammation which is also noted in respiratory diseases characterized by dyspnoea [35, 36]. Inflammation may lead to depression via activation of the indoleamine-2,3-dioxygenase enzyme [37]. When this enzyme is activated, a decrease in the production of serotonin occurs along with increases in production of kynurenic and quinolinic acids [38]. Increased production of kynurenic and quinolinic acid promotes an increase in release of glutamate resulting in decreased production of trophic factors that are associated with depression [38]. Regardless of causality, dyspnoea is reported to be clinically under-recognised which may be due to the interrelated nature of anxiety, depression and severe asthma pathophysiology and symptoms and this should be assessed as part of the multidimensional assessment of a person with severe asthma.

Our results found that dysfunctional breathing is also associated with combined anxiety and depression. Although previous literature is limited [39, 40], our results are similar to those reported by Denton and colleagues where participants with difficult asthma and dysfunctional breathing were 3.3 times more likely to have anxiety and 2.8 times more likely to have depression [41]. When faced with emotionally challenging situations, abnormal psychophysiological responses are noted in patients with dysfunctional breathing [42]. Dysfunctional breathing stimulates noteworthy changes in respiratory rate, breath-holding time, and depth of breathing that are primarily mediated by anxiety and depression [41] potentially increasing both Nijmegen scores and HADS scores. Previous literature reveals that breathing retraining improved Nijmegen scores, but has no effect on HADS scores [41] suggesting that symptoms of anxiety and depression and dysfunctional breathing should be individually assessed and treated in people with severe asthma [43].

We also highlight associations between high BMI and symptoms of both anxiety and depression. Increased BMI has been previously reported in people with severe asthma compared to people with controlled asthma [43] and healthy controls [44]. Likewise, clinical and community-based cross-sectional studies (irrespective of methodological variability) have consistently shown a relationship between obesity, anxiety and depression [45–47]. Our findings may be explained by inflammatory processes including: (1) the link between depression and low-grade chronic systemic inflammation [48], and (2) that anxiety has been associated with increased production of pro-inflammatory cytokines [49] and (3) that hs-CRP is elevated in obese people [50]. Depression may lead to inflammation mediated by weight gain [37]. CRP production is stimulated by the expansion of adipose tissue that increases synthesis of leptin, which in turn increases levels of IL- 6 [51]. Furthermore, obesity has mechanical effects on lung function, it leads to a systemic proinflammatory state, thereby increasing airway inflammation, and is associated with a number of comorbid factors [52]. Likewise, anxiety and depression may lead to elevated levels of circulating cortisol which is also observed in obesity [53]. Studies have also demonstrated that obese persons experience substantial impairments in QoL as a result of their obesity, with greater impairments associated with greater degrees of obesity [54]. To improve QoL, obesity should be assessed and managed effectively in severe asthma.

Despite having anxiety or depression alone or combined symptoms of anxiety and depression, our data shows a strong correlation between poorer HRQoL and asthma control. Elevated levels of burden may be observed in people with severe asthma due to frequent acute attacks and multiple comorbidities [4]. Anxiety and depression also correlate with level of asthma control [55] and may increase the risk of acute asthma attack. HRQoL in severe asthma is frequently impaired and is a hallmark of severe disease [56]. As HRQoL is defined as a multidimensional concept that includes domains related to physical, mental, emotional and social functioning [57], negative impacts are reported to include difficulties in relationships, stigma, and low satisfaction with life [58] highlighting the need to screen and treat symptoms of anxiety and depression in severe asthma populations.

Our findings highlight the importance in screening psychological status in severe asthma even when symptoms are not directly reported. Although not highly supported, the use of clinical measures as well as self-report questionnaires may be useful in distinguishing between dysfunctional breathing, dyspnoea and symptoms of anxiety, highlighting an area for further research. Routine screening and evaluation of psychological and associated clinical characteristics should be part of treatments promoting the right treatment for the right individual. These characteristics may be important in the development of multi-component treatment programmes that address anxiety and depression in severe asthma.

Our study is novel as it uses a multidimensional assessment to explore anxiety and depression in severe asthma and allows determination of the relationship between these symptoms and other important clinical characteristics. Our inclusion criteria focused on symptoms of anxiety and depression rather than a formal diagnosis allowing wider inclusion of participants. This may be transferred in the clinical setting where patients who do not have a formal diagnosis can still be assessed and managed. Limitations of our study include its cross-sectional design, which did not allow assessment of transience in HADS score, nor does it allow us to infer causality within our findings. One limitation of this study is that all possible confounding variables were not measured in the study. The study measured smoking status, cardiovascular disease and asthma medication treatment adherence, however no differences were found between the comparison groups. The study did not measure socioeconomical status, treated/uncontrolled anxiety or depression. A longitudinal study with a larger sample would be beneficial and would increase reliability of study results. A longitudinal study could also predict long- term associations of treatable traits in people with severe asthma with symptoms of anxiety and depression. Having evidence of long-term associations will allow for personalised assessment and management of serve asthma and co-existing comorbidities resulting in reduced HRQoL. This could be achieved by reassessing the study population longitudinally after their initial study visit using a multidimensional assessment examining airway, comorbidity, risk factors and behavioural characteristics to establish if baseline symptoms of anxiety and/or depression predict adverse asthma outcomes. Future comparative studies investigating influences of symptoms of anxiety and/or depression in people with mild to moderate asthma compared to people with severe asthma are warranted. Additionally, the Nijmegen Questionnaire was developed to assess hyperventilation syndrome which has an association with anxiety. Despite this, the Nijmegen Questionnaire is commonly used in assessment of people with severe asthma.

## Conclusion

This study found associations between dysfunctional breathing and symptoms of anxiety, dyspnoea and symptoms of depression, and associations between dysfunctional breathing, obesity and combined symptoms of anxiety and depression. Given the prevalence of anxiety and depression in this severe asthma population and its significant relationship to health-status and asthma control we recommend that symptoms of anxiety and depression be included as a standard assessment of severe asthma and that interventions to effectively manage these symptoms are explored. This will allow for effective and tailored management in individuals with severe asthma. We highlight the importance of assessment and management of extra-pulmonary characteristics in the management of severe asthma and their interrelationship. However, further research is needed to understand the underlying physiological and psychological mechanisms to distinguish breathlessness and dysfunctional breathing in severe asthma.

## Data Availability

All data generated or analyzed during the current study are included in this published article.

## Abbreviations

6MWT: 6-Minute walk test
ACQ: Asthma Control Questionnaire
AQLQ: Asthma Quality of Life Questionnaire
BMI: Body mass index
COPD: Chronic obstructive pulmonary disease
CRP: C-reactive protein
FeNO: Fractional exhaled nitric oxide
FEV_1_: Forced expiratory volume in one second
FVC: Forced vital capacity
HADS: Hospital Anxiety and Depression Scale
HRQoL: Health-Related Quality of Life
ICS: Inhaled corticosteroids
IgE: Immunoglobulin
IL-6: Interleukin-6
mMRC: Modified Medical Research Council Dyspnea Scale
NQ: Nijmegen Questionnaire
OCS: Oral corticosteroids
QoL: Quality of Life
